# Application of Design of Experiments to the Analysis of Fruit Juice Deacidification Using Electrodialysis with Monopolar Membranes

**DOI:** 10.3390/foods11121770

**Published:** 2022-06-15

**Authors:** Marcello Fidaleo, Giordana Ventriglia

**Affiliations:** Department for Innovation in Biological, Agro-Food, and Forest Systems, University of Tuscia, Via San Camillo de Lellis, 01100 Viterbo, Italy; giordy.ve@hotmail.com

**Keywords:** deacidification, citric acid, monopolar ion-exchange membranes, factorial design, electrodialysis

## Abstract

Despite the beneficial health effects of fruit juices, the high content of organic acids and low pH of some of them limit their consumption. The aim of this work was to study the deacidification of fruit juices using electrodialysis (ED) with monopolar membranes. Aqueous solutions of citric acid were used in ED deacidification experiments following a factorial design with citric acid concentration and electric current varying in the ranges of 5–25 g/L and 0.5–1 A, respectively. The design runs were characterized by a constant Faraday efficiency of 0.37 ± 0.03, suggesting that the triple-charged citrate ion (Cit^3−^) carried the electric charge through the anionic membranes. During deacidification, the pH increased in agreement with the decreasing concentration of the acid. Deacidification of pineapple juice or pineapple juice enriched with 20 g/L of citric acid using ED led to similar values of the Faraday efficiency, confirming that Cit^3−^ is the main ion migrating through the anionic membrane. However, the decrease in titratable acidity during ED treatment was accompanied by a decrease in pH. Such behavior, already reported, was explained by considering proton generation during the transformation of the single (H_2_Cit^−^) and double-charged (HCit^2−^) citrate ions into the triple-charged ion (Cit^3−^) when entering the anionic membrane.

## 1. Introduction

Electrodialysis (ED) is a unit operation for the separation or concentration of ions in solutions based on their selective electromigration through semi-permeable cationic (C) and anionic (A) membranes under the influence of an electric field [1]. In addition to homopolar membranes (C and A membranes), electrodialysis stacks can also host bipolar membranes (B), which are membranes dissociating water molecules in protons (on the cationic side of the B) and hydroxyls (on the anionic side of the B) under the effect of an electric field. The use of ED is increasing in the agri-food industry thanks to the advantages associated with its low energy consumption, efficiency, ease of use and low thermal damage to the product. In the fruit juice industry ED can be applied for deacidification, desalting, and enzyme inhibition [1]. Conventional juice deacidification methods involve the addition of additives such as sugar, CaCO_3_ or the use of ion exchange resins. However, these methods can affect the sensory and functional properties of beverages and cause health problems [2]. ED was studied in the deacidification of juices from several fruits such as cranberry [2,3,4], mandarin orange [5], passion fruit [6,7,8,9,10], tropical fruits (passion fruit, naranjilla, araza, mulberry) [7,11,12], and pineapple [13]. Deacidification using ED was achieved by applying several process configurations with two or three compartments using a combination of cationic, anionic, bipolar and ultrafiltration membranes. The aim of the process is the removal of the acid (citric, malic, quinic, etc.) from the juice. This is accomplished by selectively removing the acid anion and the H^+^ cation from the juice or by removing the anion and substituting it with OH^−^ either generated from a bipolar membrane or donated from a KOH or NaOH solution circulating in a compartment adjacent to the juice. Basically, four ED membrane stack configurations were studied for the deacidification of fruit juices: two-compartment conventional homopolar membrane electrodialysis using alternating cationic and anionic membranes (ED2AC) or just anionic membranes (ED2AA); three-compartment monopolar electrodialysis with repeating cells made up of two anionic membranes delimitated by two cationic ones (ED3CAAC); two-compartment homopolar/bipolar electrodialysis using alternating anionic and bipolar membranes (ED2AB). Substitution of the anionic membrane with an ultrafiltration membrane (U) in the different configurations was also studied.

A critical role in all the systems employing electrodialysis is represented by the anion exchange membrane, which facilitates the removal of organic anions present in fruit juices, such as citrate, malate, and tartrate anions.

In this work, we resorted to the theory of ampholyte transport through electrodialysis membranes to elucidate the behavior of anion exchange membranes in fruit juice deacidification. To this end, we considered the ED2AC configuration and used citric acid and pineapple juice as a model system. The new understanding of the behavior of the anionic membrane allowed us to explain why the ED2AC configuration leads to deacidification of fruit juices without pH increase.

## 2. Materials and Methods

### 2.1. Raw Materials

Aqueous solutions of citric acid were prepared by dissolving anhydrous citric acid in deionized water to obtain concentrations in the range of 325 g/L. These solutions were used to study the effect of citric acid concentration on pH and conductivity (k) and as feeds for the diluting, concentrating and electrode rinsing compartments in electrodialysis deacidification experiments.

For the electrodialysis tests on juice, a pulpy pineapple juice was used. The product contained only juice, not from concentrate, as the only ingredient and was purchased from a local supermarket (Conad, Bologna, Italy). The label stated the caloric and carbohydrate content as 50 kcal and 12 g of carbohydrate per 100 mL of product, respectively. Juices from the same production batch were selected.

### 2.2. Analytical Methods

Samples of model solutions prepared for calibration purposes or withdrawn during deacidification experiments were subjected to pH, conductivity, and titratable acidity measurements (TA). Samples of fruit juice withdrawn during deacidification experiments were additionally subjected to measurement of soluble solids content (SSC).

pH was measured using a multi-parameter instrument (model InoLab pH/Cond. 740, Xylem Analytics Germany Sales GmbH & Co. KG, WTW, Weilheim, Germany)) set at 20 °C. The instrument was connected to a pH probe with built-in temperature measurement (model SenTix81 PLUS, Xylem Analytics Germany Sales GmbH & Co. KG, WTW, Weilheim, Germany). Calibration for pH was performed using standard solutions of pH 2, pH 4, and pH 7.

Electrical conductivity was measured at 20 °C using a benchtop conductivity meter (InoLab Con Level 1, Xylem Analytics Germany Sales GmbH & Co. KG, WTW, Weilheim, Germany) to which a conductivity cell (model Tetracon 325, Xylem Analytics Germany Sales GmbH & Co. KG, WTW, Weilheim, Germany) was connected. The instrument was calibrated using standard solutions with conductivities of 12.88 mS/cm and 1413 µS/cm (HI 70030 and HI7030, Hanna Instruments Italia, Ronchi di Villafranca Padovana, Italy).

Total soluble solids of juice samples were determined using an electronic refractometer (model WM-7 Atago, Tokyo, Japan).

Determinations of the titratable acidity of juice samples were carried out using an automatic titrator (model Flash Steroglass, Perugia, Italy) that had two probes, one for pH and one for temperature. A 0.25 M NaOH titrant solution was initially prepared. Following initialization of the instrument and calibration using pH standards (4 and 7), testing was started. For the analysis, samples were diluted 1 to 5 with water by adding deionized water to 10 mL of each sample up to a final volume of 50 mL. Titration was stopped when a pH value of 8.1 was reached. Titratable acidity was expressed in g/L of citric acid.

The accuracy and precision of all analytical methods was checked using aqueous solutions of citric acid, conductivity and pH calibration standards and reference literature values for citric acid physicochemical properties (electric conductivity).

### 2.3. Description of the Electrodialysis System

A laboratory-scale electrodialysis plant (model EUR2, Eurodia Industrie SA, Wissons, France) was used for the deacidification tests with model solutions and fruit juice.

The ED stack of the plant consisted of 7 desalination cells (N_cells_) made up of 8 cationic (Neosepta CMX-Sb) and 7 anionic (Neosepta AMX-Sb) membranes (Tokugama Soda Co., Tokyo, Japan), separated by spacer gaskets (0.7 mm thick) and arranged in parallel between a platinum electrode (anode) and a titanium electrode (cathode). The ED stack was connected to a direct current generator (model N5767A, Agilent Technologies Inc., Santa Clara, CA, USA), which provided voltage (E) and current (I) in ranges of 0–60 V and 0–25 A, respectively. Two PVC tanks with a capacity of 1.6 L were used to hold the diluting and concentrating solution and a larger PVC tank with a capacity of 2 L was used to hold the electrode rinsing solution. The diluting and concentrating tanks were each equipped at their top with a 2 m high plexiglass tube with a metric scale to accurately measure the variation in the volume of liquid they contained. An ultrasonic distance sensor (model Tough Sonic 14, Senix Corporation, VT, USA) was inserted into the upper cavity of the concentrating tank tube to measure the liquid level in the reservoir, from which the corresponding liquid volume was estimated.

The plant was equipped with three polypropylene centrifugal pumps that allowed the recirculation of solutions inside the stack, with a nominal capacity of 0.25 m^3^/h and a total discharge head of 15 m of water.

The flow rates of solutions circulating in the diluting and concentrating tanks were adjusted using ball valves, in the range of 90–110 L/h, taking care to keep them almost similar between the two compartments.

For the electrode solution, the flow rate was set to 300 L/h.

To measure the conductivity and temperature of the solutions in the plant, three conductivity cells were used: two flow cells (model Tetracon DU/T, Xylem Analytics Germany Sales GmbH & Co. KG, WTW, Weilheim, Germany)) used for diluting and concentrating solution tanks, and one discontinuous cell (model Tetracon 325, Xylem Analytics Germany Sales GmbH & Co. KG, WTW, Weilheim, Germany ) for the electrode rinsing fluid.

Three instruments were used to measure conductivity and temperature data: two InoLab Con Level 1 devices (Xylem Analytics Germany Sales GmbH & Co. KG, WTW, Weilheim, Germany) and the multi-parameter instrument InoLab pH/Cond. 740 (Xylem Analytics Germany Sales GmbH & Co. KG, WTW, Weilheim, Germany).

The process temperature was regulated and controlled using a thermocryostat (model PL1, Lauda Master, Lauda-Königshofen, Germany) equipped with a thermocouple inserted into the electrode rinsing solution tank. The thermocryostat provided cooling water flowing inside coil heat exchangers inserted into the three tanks.

An application for the control of the ED unit (EDCP) developed in LabVIEW (National Instruments Italy S.R.L, Assago, Italy) facilitated control of the electric generator and acquisition and storage of real-time data using probes installed on the ED system and from manual operation. Parameters were acquired at an acquisition rate of 1 Hz (one acquisition per second).

### 2.4. Experiments with the ED Plant

Before using the system for the electrodialysis process, the stack was disassembled to observe the wear and condition of the membranes. Once reassembled, the membranes were washed with deionized water. Subsequently, washes were performed alternating deionized water with hydrochloric acid solution (HCl, 1 mol/L) and sodium hydroxide solution (NaOH, 3 g/L). Finally, the system was subjected to multiple washes with deionized water to ensure effective cleaning of residues from the HCl and NaOH solutions. The ED plant was used to perform limiting current intensity tests and deacidification experiments with model solutions of citric acid or fruit juice.

#### 2.4.1. Limiting Current Intensity Tests

Limiting current intensity tests were carried out to establish the maximum current intensity to which the membranes could be subjected without being damaged, the so-called limiting current intensity (I_lim_). This parameter made it possible to identify the current at which to perform the deacidification tests, which had to be lower than I_lim_. In order to keep the concentration of the solutions flowing in the C and D compartments approximately the same, the tubes of D and C connected to the stack were reversed. A solution of citric acid with a concentration of 25 g/L was used as an electrode rinsing solution while in the concentrating and diluting tanks a solution of 25 g/L was initially used for the first experiments and then its concentration was reduced by removing an aliquot of solution and replacing it with deionized water to vary its electric conductivity from approximately 3.15 mS/cm to 437 µS/cm.

The EDCP program was used to perform the tests. Eight tests consisting of three replicates each were performed. The voltage (E)-current (I) data were automatically saved on the computer and used to calculate I_lim_. The limiting current intensity was determined from the E-I response of the ED stack using the Cowan and Brown plot [14], a graph in which the E/I is reported as a function of 1/I. The current value corresponding to the minimum on the Cowan and Brown diagram was chosen as the limiting current value (I_lim_).

#### 2.4.2. Deacidification Experiments with Citric Acid Solutions

The tests were conducted in batch mode at a temperature of 20 °C, continuously recirculating the diluting and concentrating solutions. All deacidification experiments were carried out at an electric current intensity lower than the limiting one (I_lim_).

Before each test, the system was emptied of the liquid left over from the previous test and several washes with deionized water were performed until a conductivity between 200 and 600 µS/cm was reached in the tanks. The tanks were filled with citric acid solutions, using 1.9 L, 1.8 L and 2 L for the diluting, concentrating and electrode rinsing solutions, respectively. The concentrations of the solutions used for the concentrating and electrode rinsing solution tanks were kept constant for all tests, 5 g/L and 25 g/L, respectively.

The tests were conducted following a 2 × 2 factorial design added with a center point replicated three times. The two factors of the experiments were the initial concentration of the solution present in the diluting tank (C_D_) and the intensity of the electric current (I). Their low, middle and high levels are reported in Table 1.

The level of the initial concentrations in the diluting tank in the various tests carried out differed from that indicated in Table 1 due to the residual volumes of solution inside the plant which modified the starting concentrations. The experimental conditions adopted are shown in Table 2.

After starting the pumps for recirculation of the solutions, a few minutes’ wait allowed the solutions just inserted into the tanks to mix completely with the residual liquid and the temperature, controlled with the cryothermostat, to reach the desired value of 20 °C. The test was then started by turning on the current generator using the EDCP program. Several data were acquired by the program automatically or read on the instrument displays and entered manually through the graphical interface every two minutes. A sample of solution (20 mL) was taken every 10 min from the diluting stream and used to measure conductivity and pH.

The duration of each test was a function of the initial concentration of citric acid and varied according to the current intensity applied. In each test, deacidification was carried out until the final conductivity of the diluent solution reached 1.4 mS/cm, except in one case.

The efficiency of the deacidification process using ED was characterized in terms of Faraday efficiency (Ω). It relates the mass of electrolyte that is transported across membranes from diluting to concentrating compartments during the deacidification test, expressed in moles (∆n_B_; ∆n_BD_ or ∆n_BC_ for the diluting or concentrating solution, respectively), to the amount of charge provided by the generator, expressed in moles of elemental charge (n_F_):

(1) $$\Omega =\frac{\Delta n_{B}}{n_{F}}$$

where n_F_ can be calculated based on the following expression:

(2) $$n_{F}=\frac{N_{\mathrm{cell}}\mathrm{It}}{F}$$

and N_cell_ is the number of ED cells used, I is the electric current intensity, F is the Faraday constant (=96,485.3 C/mol) and t is the process time.

#### 2.4.3. Deacidification Experiments with Fruit Juice

Before using the juice in the ED plant, it was clarified by filtration to remove the pulp and limit fouling problems. In fact, for pulpy juices it is suggested that deacidification using ED is carried out on the filtered or centrifuged juice [10]. Filtration was carried out using a vacuum filtration device employing a pump (model UN840 KNF Neuberger Laboport, Germany) and a filter (model WHA-10370319, Whatman, UK). The filtered juice was introduced into a two liter flask and its conductivity and pH were measured.

Two deacidification tests were performed under the same flow rates and temperature as the tests conducted with the citric acid solutions. For the first test the filtered fruit juice and an electric current intensity of 0.75 A were used. For the second test, the filtered juice was enriched with citric acid (40 g of citric acid were brought to a final volume of 2 L using the fruit juice) and the applied electric current was 1 A.

For the concentrating or electrode rinsing solutions a volume of 1.8 L or 2 L and a citric acid solution of 5 g/L or 25 g/L were used, respectively.

Once the pumps were turned on, the generator turned on, and the EDCP program set, the deacidification test was started. Data were recorded using the EDCP program both automatically from the sensors or manually every 2 min.

Samples of 20 mL were withdrawn from the diluting tank every 5 min (first trial) or at varying times (second trial) using a Falcon tube and placed in a refrigerator for subsequent analysis.

During deacidification, in addition to the liquid level and conductivity measurements from online sensors, the pH was measured automatically thanks to the multi-parameter instrument InoLab pH/Cond. 740 (Xylem Analytics Germany Sales GmbH & Co. KG, WTW, Weilheim, Germany) that allowed connection with a pH probe with built-in temperature measurement (model SenTix81 PLUS, Xylem Analytics Germany Sales GmbH & Co. KG, WTW, Weilheim, Germany). A juice recirculation system was constructed consisting of a tube (internal diameter of 3 mm and length of 1.50 m) that used a peristaltic pump (model 7518-10, Masterflex easy load, Cole-Parmer North America, Vernon Hills, IL, USA) to move the juice from the diluting tank and, before reintroducing it into the tank itself, sent it through a glass flow cell (model D01/T, Xylem Analytics Germany Sales GmbH & Co. KG, WTW, Weilheim, Germany) that housed the pH probe (model SenTix81 PLUS, Xylem Analytics Germany Sales GmbH & Co. KG, WTW, Weilheim, Germany).

### 2.5. Statistical Methods

Experimental design and univariate and multivariate regression analysis were carried out using JMP statistical software package v. 16.1.0 (SAS Institute, Cary, NC, USA).

## 3. Results

### 3.1. Chemico-Physical Properties of Citric Acid Solutions

**Figure 1:**
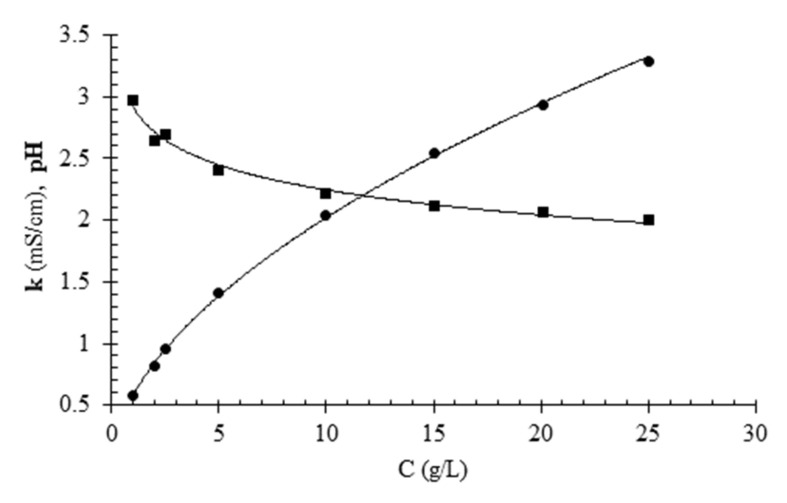
Electric conductivity k (●) and pH (∎) at 20 °C of citric acid aqueous solutions as a function of citric acid concentration (C). The symbols refer to experimental data while the continuous lines refer to approximating curves.

The pH and the electric conductivity of solutions are important properties in deacidification treatments using ED. The first parameter is directly linked to the hydrogen ion concentration while the second one is correlated to the concentration of electrolytes in solution. Figure 1 shows the pH and electric conductivity of aqueous citric acid solutions as a function of concentration. As can be observed, as the concentration increases, the pH decreases, as expected considering that citric acid is a weak triprotic acid. In the concentration range 5–25 g/L, corresponding to the interval of concentration of the model solutions used in deacidification experiments, the pH varied in the range 2.4–2.0. The concentration of citric acid had a positive effect on the electrical conductivity of the solution, as is observed for all electrolytes, at least up to a critical concentration beyond which conductivity begins to decrease. The conductivity-concentration data were used to derive the coefficients of a third-order polinomial equation of concentration vs. conductivity that was used to indirectly estimate citric acid concentration from conductivity measurements for citric acid model solutions during ED.

### 3.2. Physical-Chemical Properties of Pineapple Fruit Juice

The properties determined for the pineapple juice as such, after citric acid addition and during deacidification were titratable acidity (TA), pH, conductivity and total soluble solids. The pineapple juice used in this work had the following chemical and physical characteristics: pH = 3.8 ± 0.1, AT = 5.6 ± 0.8 g/L, k = 2.94 ± 0.05 mS/cm, SSC = 11.7 ± 0.1 °Bx. For the citric acid enriched juice the following values were obtained: pH = 2.7 ± 0.1, AT = 27 ± 2 g/L, k = 3.44 ± 0.04 mS/cm, and SSC = 13.5 ± 0.1 °Bx. The addition of citric acid resulted in a decrease in pH and an increase in AT. However, the pH that was reached was higher than that corresponding to a citric acid solution with a concentration of 27 g/L (approximate pH = 2, as observed from Figure 1). This suggests that the juice had some buffering power due to the presence of other ions in solution such as the potassium ion, as pointed out already [13]. The measured parameters agree with a study [15] that analyzed major and some minor constituents for a series of fresh pineapple juices: soluble solids 11.2–16.2%, TA (reported as citric acid) 4.6–12.1 g/L, fructose 17.2–47.5 g/L, glucose 12.1–45.2 g/L, sucrose 24.7–97.3 g/L, citric acid 4.39–11.51 g/L, malic acid 0.73–3.91 g/L, isocitric acid 0.80–0.265 mg/L, and potassium 0.830–1.410 g/L. From the study it can be assumed that the main acid and cation of pineapple juice are respectively citric acid and potassium.

### 3.3. Limiting Current Tests

**Figure 2:**
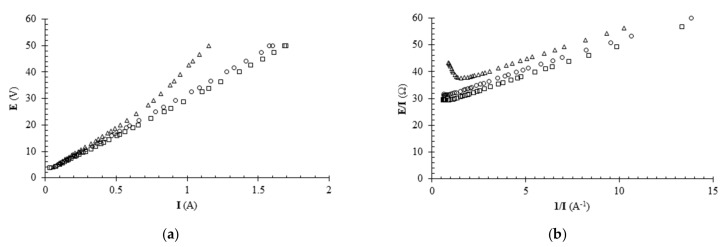
Voltage-current (E-I) curves (**a**) and corresponding Cowan and Brown plot (**b**) for aqueous citric acid solutions at three different citric acid concentrations (∆ = 0.9 g/L; ○ = 3.7 g/L; □ = 23.9 g/L).

Figure 2a shows the results of the limiting current tests. The voltage applied to the stack showed a linear trend for low values of the current. For higher values, the voltage deviated from the line approximating the first linear zone, and this deviation occurred for lower current values the lower the concentration (or corresponding conductivity) of the solution. This behavior is consistent with previous results obtained using strong electrolytes [16,17] or the sodium salts of monoprotic, diprotic and poliprotic acids [18,19,20,21,22]. To facilitate the determination of the limiting current, the experimental voltage-current data were plotted on a Cowan and Brown diagram (Figure 2b).

**Figure 3:**
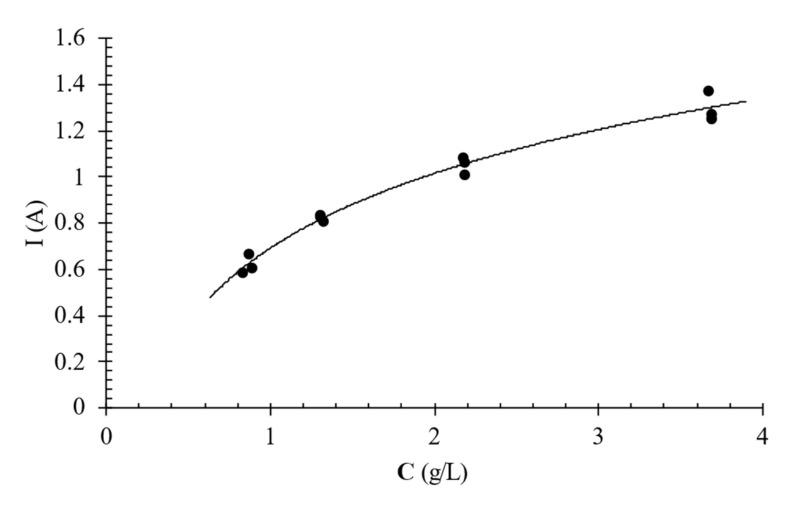
Limiting current intensity (I_lim_) vs. solution citric acid concentration (C). The symbols refer to experimental data while the continuous lines refer to the approximating curve.

For each data set on Figure 2b, the abscissa of the minimum point was determined from which the limiting current was calculated. The values thus determined are shown as a function of acid concentration in Figure 3. The maximum I_lim_ value determined was 1.3 A at a concentration value of 3.7 g/L. For higher acid concentration values, it was not possible to find the limiting current intensity because the corresponding data did not show a relative minimum point on the Cowan and Brown diagram. However, it can be stated that the limiting current for higher concentrations is definitely greater than 1.3 A. Since the acid concentration did not drop to values below 3.7 g/L during the desalination tests, currents lower than 1.3 A were applied in the range of 0.5–1 A as a precaution.

### 3.4. Deacidification Tests

**Figure 4:**
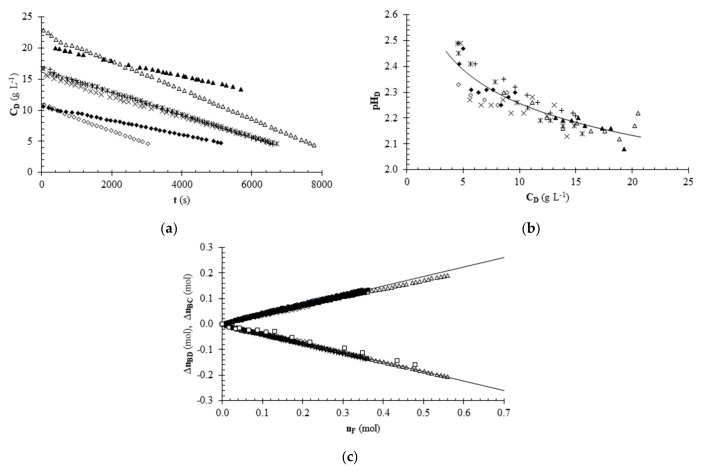
Deacidification experiments of citric acid solutions at different electric current intensities (I = 0.5 A, open symbols; I = 1 A, closed symbols; I = 0.75 A, x, +, ∗) and initial concentrations (C_D0_ = 25 g/L, ▲, ∆; C_D0_ = 12.5 g/L, ◆, ◊; C_D0_ = 18.75 g/L, x, +, ∗): (**a**) citric acid concentration (C_D_) vs. time (t); (**b**) pH vs. citric acid concentration (C_D_); (**c**) variation in the number of moles of citric acid or titratable acidity for the diluting (∆n_BD_) and concentrating (∆n_BC_) tank vs. the number of moles of elemental charge (n_F_) (symbols ○ and □ refer to pineapple fruit juice and to pineapple fruit juice enriched with citric acid, respectively). Symbols refer to experimental data while the continuous lines refer to approximating curves.

Figure 4a–c show the main results of the deacidification tests with citric acid aqueous solutions carried out according to the experimental plan described above. The data shown in Figure 4a represent the concentration of citric acid of the solution held in the diluting tank as a function of time. The data sets are grouped into three groups: the first group, at the bottom of the plot, is made up of the two tests carried out at a low initial acid concentration; in the middle part of the plot, there is the group relating to the central test of the experimental plan, corresponding to an intermediate initial concentration; the last group, at the top of the plot, represents the two tests carried out at a higher initial acid concentration. The effect of the electric current on this diagram corresponds to the different slope of the curves, which corresponds to the deacidification rate: the higher the electric current the higher the deacidification rate. Overlapping data from the central trial of the experimental plan highlight good reproducibility of the experiments.

Figure 4b shows pH data measured as a function of citric acid concentration on samples taken during different deacidification trials. The pH data appear to be negatively correlated with the concentration of the solutions. This is in line with theory, i.e., a solution with higher concentration of citric acid has a lower pH, since it is an acidic substance. The pH values observed during desalination are very close to those that can be predicted from Figure 1; i.e., the values corresponding to standard solutions of citric acid. The experimental pH data regarding samples taken during desalination were more variable and required longer times for the glass electrode to stabilize than the standard solutions. In conclusion, it can be said that ED enabled a reduction in the concentration of citric acid solutions and this reduction corresponded to an increase in pH.

From the volumes of the solutions present in the ED system and the concentration data of the solutions obtained from the conductivity data, it was possible to calculate the variation with respect to the initial time of the number of moles of acid present in the diluting (∆n_BD_) and concentrating (∆n_BC_) tanks as a function of time for each deacidification test. Figure 4b reports these values as a function of the number of charge equivalents transferred across the membranes (n_F_). As can be seen, the number of moles transferred from one compartment to another varied linearly as a function of n_F_, with slopes of opposite signs, but similar absolute value for diluting and concentrating tanks. From the analysis of all the experimental data, using the method of least squares and Equation (2), it was possible to estimate as the mean value and standard deviation of the slopes in absolute value, the following value, corresponding to the Faraday efficiency: 0.37 ± 0.03. This value is in line with the hypothesis of ideal membranes and the assumption that the Cit^3−^ anion and the H^+^ cation carry the electrical charges through the anionic and cation membrane, respectively. It should be noted that in the pH range relative to the desalination tests (2.0–2.4), citric acid was present in the undissociated form (approximately 80%) and in the H_2_Cit^−^ form (approximately 20%) while the other forms (HCit^2−^ and Cit^3−^) are present in a much lower percentage [23].

### 3.5. Analysis of the Faraday Efficiency

The experimental plan used for the deacidification tests allowed the study of the main effects and interactions of the initial acid concentration and the electric current on the Faraday efficiency. In fact, it was a 2 × 2 factorial plan added with a replicated central point. The presence of the central point allowed verification of the presence of possible curvature effects and at the same time to testing of the statistical significance of the effects through replications.

Table 3 shows the Faraday efficiency calculated for the diluting compartment (Ω_D_) as a function of factor levels for all deacidification tests.

Data were analyzed using a linear regression model:

(3) $$y=b_{0}+b_{1}x_{1}+b_{2}x_{2}+b_{12}x_{1}x_{2}$$

where *x*_1_ and *x*_2_ represent the levels of the coded C_D_ and I factors, i.e., expressed on a scale where −1, 0, and +1 correspond to the low, middle, and high values of the factor level, respectively. Table 3 reports estimates of the unknown coefficients, the corresponding standard error, and the *p*-values related to the hypothesis tests on the significance of the coefficients.

Regression analysis of the Ω_D_ data showed that neither factor had a significant effect at a significance level α = 1%. The only significant term was the intercept which coincides with the Faraday efficiency; constant, therefore, and independent of initial acid concentration and electric current.

### 3.6. Fruit Juice Deacidification Tests

**Figure 5:**
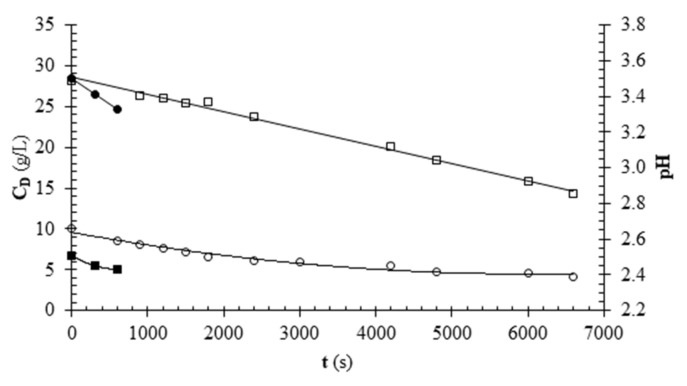
Time course of pH (●, ○) and titratable acidity concentration (C_D_) (∎, □) for the deacidification of pineapple juice carried out at I = 0.75 A as such (closed symbols) or at I = 1 A enriched with approximately 20 g/L of citric acid (open symbols).

Figure 5 shows the results of fruit juice deacidification tests conducted at I = 0.75 or 1 A in terms of titratable acidity and pH. The titratable acidity decreased during the test due to the migration of citric acid. The variation in the number of moles of titratable acidity (data obtained from the titratable acidity) as a function of the number of moles of electric charge transferred (n_F_) are reported in Figure 4c together with the data from citric acid solutions deacidification experiments. The data obtained for the fruit juice are close to those for the tests with model solutions. The pH for the trial performed at I = 0.75 A showed a decrease as a function of time. For the trial at I = 1 A, the pH underwent a slight change as a function of time, decreasing initially, then reaching a minimum and subsequently starting to increase towards the end of the test. A decrease in the pH of the ED treated juice was also observed by other authors [6,13].

In the case of pineapple juice [13], a reduction in TA from 6.7 to 6.0 g/L corresponded to a decrease in pH from 4.01 to 3.75. The pH reduction was attributed to the buffer salts present in the juice, their ionization and removal during the process. The deacidification of passion fruit juice, whose acidity is mainly due to citric acid, using conventional ED, led to similar results [6]. For a decrease in titratable acidity from 43 to 37 g/L, the pH decreased from 2.9 to 2.4. The pH decrease was attributed to the extraction of ionic species, both protons and H_2_Cit^2−^, enhancing the dissociation equilibrium of the weak acid and, consequently, impacting the free acidity.

Without entering into the merits of whether electrodialysis configuration is better suited for fruit juice deacidification, it is worth mentioning that the sourness of organic acids is influenced by the concentration of acid and pH, not only pH [24,25]. In fact, the pH of the abovementioned ED treated pineapple juice decreased from 4.01 to 3.75, and its tartness, as measured using a sensory test, decreased from 4.1 to 2.9 on a scale from 1 to 5 (with 3 corresponding to fair), thus improving the overall acceptability of the juice.

## 4. Discussion

The experiments carried out in this work facilitate for the first time a better understanding of the deacidification mechanism of citric acid solutions using conventional ED in light of recent research findings. The statistically designed deacidification experiments showed that the Faraday efficiency was independent of the electric current intensity and acid concentration used and equal to approximately 1/3, indicating that the triple-charged anion Cit^3−^ migrated through the anionic membrane. Citric acid is an anpholyte, i.e., a substance with a chemical structure and electrical charge that depend on the pH of the medium due to its participation in protonation-deprotonation reactions. In the pH range of the citric acid solutions used in ED experiments, approximately 2.0–2.5, the undissociated form of citric acid accounts for approximately 95 to 80%, the remaining being H_2_Cit^−^, as can be roughly estimated from the citric acid dissociation diagram [23]. It was recently demonstrated with reference to the ED of NaH_2_PO_4_ solutions, that transformation of one form into another takes place not only in the solution, which is located in the intermembrane space, but also inside ion-exchange membranes [26,27,28]. The transformation of an ampholyte from one form to another is thus possible when it enters or leaves the membrane. This is because the pH of the internal solution present in the membrane is different than that of the outside solution. In anion exchange membranes, the pH of the internal solutions is reported to be one to three units higher than that of the external solution because H^+^ ions are pushed out from the membrane as coions. Thus, the transport of ampholytes in systems with ion-exchange membranes is coupled with the chemical reactions of protonation-deprotonation of species entering/leaving the membrane. These considerations can be applied to citric acid migrations through anion exchange membranes. Since the feed contained only H_3_Cit and H_2_Cit^−^ species, we can assume that deprotonation reactions occurred at the anion exchange membrane/solution interface leading to the release of the triple-charged citrate ion that migrated through the anionic membrane and to the H^+^ ions that migrated through the solution in the opposite direction. Thanks to the migration of H^+^ ions through the cation exchange membranes, the overall process results in a decrease in citric acid concentration from the diluting stream leading to a corresponding increase in pH. When submitting the pineapple juice to ED, several species are present in solutions with citric acid forms and potassium as the main acid and cation respectively. In this case, what may happen is that the H^+^ ions compete with the K^+^ cations at the cation exchange membrane; thus, the H^+^ ions generated at the anion exchange membrane in the diluting compartment are not balanced by their migration through the cation exchange membrane resulting in an increase in their concentration, and a corresponding decrease in pH. Once most of the K^+^ ions are removed from the diluting compartment, the H^+^ ion migrations through the cationic membranes increase, and thus the pH starts to increase, as observed in the deacidification of pineapple juice carried out at I = 1 A.

## 5. Conclusions

In this paper, the deacidification of juices derived from fruits presenting high concentrations of organic acids, mainly citric, using monopolar membrane electrodialysis in the classical two-compartment configuration, was analyzed. Through a design of experiments approach, it was shown that ED enables a decrease in the acid concentration and a simultaneous increase in the pH of citric acid solutions. ED treatment of pineapple juice resulted in a decrease in titratable acidity without pH increase, as previously reported in a few articles. This led some researchers to consider conventional ED as ineffective in the deacidification of fruit juices. However, sourness is correlated to both titratable acidity and pH; thus, the reduction in titratable acidity, even if not accompanied by an increase in pH, can lead to fruit juices with a less perceived sourness, as already shown. The mechanism of pH decrease is explained by the theory of ampholyte transport through electrodialysis membranes. Thus, this work contributes to a better understanding of citric acid anion transport through anion exchange membranes, a process that is involved in several ED configurations proposed for fruit juice deacidification. Further research to validate the proposed transport mechanism with other organic acids/fruit juices may lead to improvements of fruit juice deacidification processes based on electrodialysis.

## Figures and Tables

**Table 1:** Factors and levels.

Factor	Levels		
	Low	Middle	High
C_D_ (g/L)	12.5	18.75	25.0
I (A)	0.5	0.75	1.0

**Table 2:** Coded and natural levels of initial concentration of the diluting solution (C_D0_) and electrical current intensity (I) together with Faraday efficiencies (Ω_D_) determined for the diluting tank for the deacidification experiments.

Run	Pattern	C_D0_ (g/L)	I (A)	Ω_D_ (−)
1	+ −	21.53	0.5	0.335
2	0 0	15.95	0.75	0.332
3	− −	10.83	0.5	0.322
4	− +	11.03	1	0.298
5	+ +	22.86	1	0.326
6	0 0	16.69	0.75	0.345
7	0 0	16.94	0.75	0.334

**Table 3:** Estimated unknown parameters of Equation (3) for Faraday efficiency (Ω_D_), together with corresponding standard errors and *p*-values. Asterisk indicates statistical significance (α = 0.05).

Term	Estimate	Error st.	*p*-Value
*b*_0_	0.3747809	0.006163	<0.0001 *
*b*_1_	0.0122336	0.008685	0.2537
*b*_2_	−0.010135	0.008151	0.3020
*b*_12_	0.0030436	0.008695	0.7494

## Data Availability

Data is contained within the article.
